# Therapeutic potential of synthetic microRNA mimics based on the miR-15/107 consensus sequence

**DOI:** 10.1038/s41417-025-00885-w

**Published:** 2025-03-22

**Authors:** Glen Reid, Marissa Williams, Yuen Yee Cheng, Kadir Sarun, Patrick Winata, Michaela B. Kirschner, Nancy Mugridge, Jocelyn Weiss, Mark Molloy, Himanshu Brahmbhatt, Jennifer MacDiarmid, Nico van Zandwijk

**Affiliations:** 1 Asbestos and Dust Diseases Research Institute (ADDRI), Sydney, NSW Australia; 2 School of Medicine, University of Sydney, Sydney, NSW Australia; 3 Institute for Biomedical Materials and Devices (IBMD), University of Technology Sydney, Sydney, Australia; 4 EnGeneIC Ltd, Sydney, NSW Australia; 5 The Australian Proteome Analysis Facility, Macquarie University, Sydney, NSW Australia; 6 Present Address: Department of Pathology, University of Otago, Dunedin, New Zealand; 7 Present Address: Department of Thoracic Surgery, University Hospital Zurich, Zurich, Switzerland

**Keywords:** Non-small-cell lung cancer, Mesothelioma, Gene delivery

## Abstract

MicroRNA expression is frequently suppressed in cancer, and previously we demonstrated coordinate downregulation of multiple related microRNAs of the miR-15/107 group in malignant pleural mesothelioma (PM). From an alignment of the miR-15 family and the related miR-103/107, we derived a consensus sequence and used this to generate synthetic mimics. The synthetic mimics displayed tumour suppressor activity in PM cells in vitro, which was greater than that of a mimic based on the native miR-16 sequence. These mimics were also growth inhibitory in cells from non-small cell lung (NSCLC), prostate, breast and colorectal cancer, and sensitised all cell lines to the chemotherapeutic drug gemcitabine. The increased activity corresponded to enhanced inhibition of the expression of target genes and was associated with an increase in predicted binding to target sites, and proteomic analysis revealed a strong effect on proteins involved in RNA and DNA processes. Applying the novel consensus mimics to xenograft models of PM and NSCLC in vivo using EGFR-targeted nanocells loaded with mimic led to tumour growth inhibition. These results suggest that mimics based on the consensus sequence of the miR-15/107 group have therapeutic potential in a range of cancer types.

## Introduction

The global repression of microRNA expression is a common feature of cancer [1], and many downregulated microRNAs have been shown to possess tumour-suppressing activity in models based on a range of cancer cell types [2]. MicroRNAs are short noncoding RNAs, 18–25 nucleotides in length, that repress gene expression through a range of sequence-dependent interactions with target sites found predominantly in the 3′UTR of mRNAs [3–6]. Individual microRNAs control the post-transcriptional gene expression of numerous cancer-related genes and can thus control the function of entire pathways. As such, microRNA-based drugs represent an attractive approach to cancer therapy, with recent approvals of siRNA-based drugs further underlining their potential [7].

Of the microRNA families dysregulated in cancer, the miR-15/16 family is one of the best characterised. The loss of expression of the miR-15a/16-1 cluster, first demonstrated in chronic lymphocytic leukaemia [8], has since been observed in a range of solid tumours, including non-small cell lung cancer (NSCLC) [9], prostate cancer [10] and pleural mesothelioma (PM) [11]. Increasing the expression of these microRNAs leads to growth inhibition in cell lines derived from these tumour types, as well as breast [12, 13] and colorectal cancer [14, 15], and has been shown to involve regulation of targets such as BCL2 [16] and multiple cell cycle [17] and growth factor signalling genes [18]. As well as the miR-15a/16-1 cluster found at the 13q14 locus, a gene duplication encoding the related miR-15b/16-2 cluster is found at 3p21, with the related miR-497/195 cluster on chromosome 17. The miR-15/16 family includes an additional 5 microRNAs, all sharing the same seed sequence (5′-AGCAGC-3′) at nucleotide positions 2–7. It has been further suggested that the family be extended to include miR-103 and miR-107, in which the same sequence is found at positions 1–6 [19].

As the members of the miR-15/107 family share the same seed sequence, there is extensive overlap in both predicted and validated targets of the individual members [17]. Despite the apparent redundancy in activity suggested by their predicted targets, it is interesting to note that the members of the miR-15/16 are frequently repressed together. This is most evident in PM, where miR-15a, 15b, 16 and 195, as well as the related miR-103 and 107, are coordinately downregulated [11]. Additional studies have revealed coordinate downregulation of the miR-15 family members miR-15a, miR-16, miR-195, miR-497 and miR-503, and the miR-103/107 microRNAs in, for example, lung [9, 20–23] and prostate cancer [10, 24–27]. This suggests that there might be subtle yet important differences in the activity of the individual members of this family, which would require all downregulated members to be restored for a therapeutic approach to be successful.

Delivery of functional nucleic acids has proven problematic due to off-target effects, but the EDV^TM^ (EnGeneIC Dream Vector) nanocell packages therapeutic concentrations of siRNA and miRNA and is targeted to deliver the payload directly into tumour cells [28]. In this study, we were thus able to investigate the possibility of using a non-natural microRNA mimic based on the consensus sequence of the miR-15/107 group to control the growth of cancer cells in vitro and in vivo. Compared with a mimic consisting of the native miR-16 sequence, these consensus mimics exhibited an enhanced ability to inhibit the growth of a range of cancer cell types and have the potential to serve as microRNA-based therapy for tumours in which the miR-15/16 family is downregulated.

## Material and methods

### Cell lines and tissue culture

The mesothelioma (H28, H2452, H2052 and MSTO-211H), NSCLC (A549, H460 and H1975), prostate cancer (PC-3 and LNCaP), breast cancer (MCF-7 and MDA-MB-231) and colorectal cancer (HCT116) cell lines were purchased from ATCC. The mesothelioma line MM05 was a gift from Dr Kwun Fong (The Prince Charles Hospital, Brisbane), and the additional mesothelioma lines VMC23, SPC111 and SPC212 were a gift from Dr Michael Grusch (Vienna Medical Centre, Austria). Cells were cultured in RPMI medium supplemented with 10% foetal calf serum and under conditions recommended by ATCC or described previously. Cell lines were periodically tested for mycoplasma contamination using the Mycoplasma Detection Kit (Thermo Scientific). Cell line identity was confirmed by STR profiling (once per cell line per year) carried out at the Australian Genome Research Facility (Melbourne, Australia) using the Promega GenePrint 10 system.

### MicroRNA mimics and transfection

**Fig. 1:**
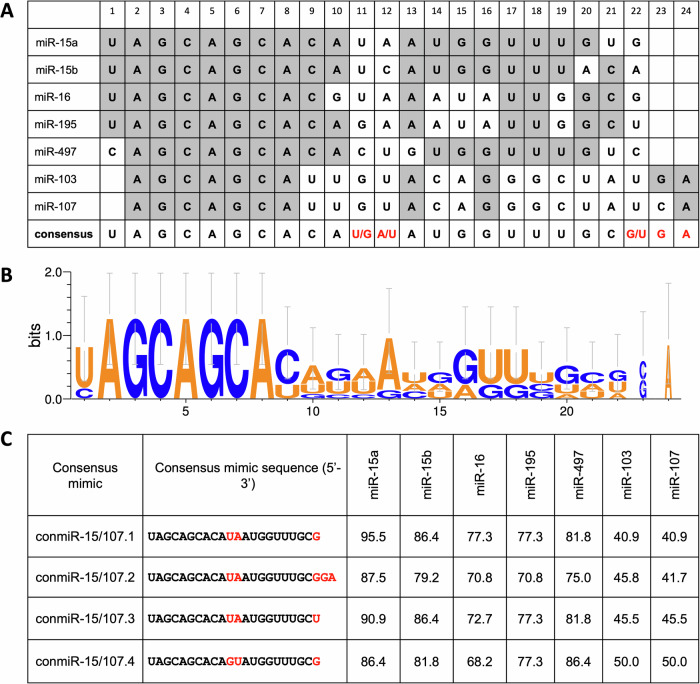
Design of mimics based on the consensus sequence of the miR-15/16 family. **A** The 5 members of the miR-15 family with detectable expression in PM samples and the related miR-103 and miR-107 were aligned. To generate a consensus sequence, positions at which one base was most frequently detected are shaded. Red denotes positions at which more than one base was equally prevalent or where less than half the aligned sequences contained that position. **B** A graphical representation of the consensus sequence logo generated with WebLogo [65]. **C** Sequences of four mimics based on the consensus sequence (bases differing between the four sequences are in red) and their individual percentage homologies to the endogenous microRNAs used to generate the consensus.

Mimics of the endogenous microRNAs miR-15a and miR-16 consisted of a mature microRNA corresponding to the sequence listed in miRBase, with a complementary passenger strand. Non-natural microRNA mimics were based on the consensus sequence of the miR-15/107 group as active strand, also with complementary passenger strand (Fig. 1). All mimics were purchased from Shanghai GenePharma. Mimics were introduced into cells by reverse transfection using Lipofectamine RNAiMAX (Thermo Scientific) as described previously [29].

### Growth inhibition and colony formation assays

Following mimic transfection, cells (2500 per well in 96-well plates) were cultured for 96 h, and proliferation was measured using a SYBR Green-based assay as previously described [29]. Alternatively, following transfection in 96-well plates, cells were transferred 24 h later into 24-well plates and allowed to form colonies for 7–14 days. Thereafter, cells were fixed, stained with crystal violet (0.1% in 10% Methanol), and colonies counted. For quantification, colonies were destained in 2% SDS and absorbance measured at 562 nm. Where described in the text, mimic transfected cells were additionally treated with gemcitabine at the concentrations indicated.

### Target gene expression

For the assessment of target gene mRNA expression, total RNA was isolated from cells transfected in 6-well plates. To generate cDNA, 250 ng total RNA was reverse transcribed using a mixture of Oligo(dT) and random primers (each at 7.5 ng/µl), and 1 µl AffinityScript reverse transcriptase in a reaction volume of 10 µl, with annealing for 5 min at 25 °C, cDNA synthesis for 15 min at 42 °C and denaturing for 5 min at 95 °C. After diluting the cDNA 1:5, 2 µl was used in each qPCR, which was carried out with 1x KAPA SYBR Green Mastermix (Kapa), forward and reverse primer (each at 180 nM; sequences were reported previously [11]) and ROX, in a total volume of 10 µl. Reaction conditions consisted of 10 min enzyme activation at 95 °C followed by 40 cycles of 15 s at 95 °C and 30 s at 55 °C. Expression levels were determined applying the 2^−ΔΔCq^ method with normalisation to 18S ribosomal RNA, expressed relative to negative control transfected cells.

### Sequential window acquisition of all theoretical fragment ion spectra (SWATH) mass spectrometry

SWATH-MS was carried out following previously reported protocols [30, 31]. Cells were transfected with mimics and controls in 6-well plates and harvested at 72 h post-transfection. Cell pellets were resuspended in 200 µL lysis buffer (0.1% sodium deoxycholate, 0.1 M triethylammonium bicarbonate) and heated to 99 °C for 5 min. After cooling to <40 °C, benzonase nuclease was added (1:10,000) and incubated for 30 min at RT to degrade DNA. Protein concentrations were estimated using a BCA protein assay kit (Pierce). An aliquot of 100 µg was reduced in the presence of 10 mM dithiothreitol for 30 min at 60 °C, alkylated in the presence of 20 mM iodoacetamide for 30 min at 37 °C and digested with trypsin overnight. The samples were acidified to quench digestion and precipitate sodium deoxycholate, spun at 14,000 rpm and lyophilised in a vacuum concentrator. Ion library generation for SWATH quantitation was carried out by online strong cation exchange coupled reversed phase liquid (RP) chromatography and ESI MS/MS on a TripleToF 5600 mass spectrometer. LC-MS/MS was performed on a pool of the consensus mimics and individual analyses of c81 and miR-16. Acquired data were searched against the human UniProt database using the paragon algorithm in ProteinPilot software version 4.2 and the search results were used as an ion library. For SWATH-MS, all cell lines were analysed in 3 injection replicates by 1 h RP SWATH-MS on a TripleToF 5600 MS using a variable window SWATH acquisition method allowing 60 m/z windows in a mass range from 400 to 1250 m/z. Windows were selected based on intensity densities from previous LC-MS/MS analyses. Acquired SWATH data were imported into PeakView software version 2.1 with SWATH microApp 2.0 to extract peptide fragment intensities using the cell lines specific ion library generated by LC-MS/MS. After processing, peptides with extraction FDR < 1% were exported and protein areas were calculated (sum of all fragment area under the curve per protein). Protein areas were imported into Perseus version 1.5 for data analysis. Protein peak areas per sample were normalised by median protein peak areas and the list of proteins was reduced to differentially expressed proteins based on ANOVA statistics (*p* < 0.01).

### Luciferase reporter assays

Fragments of the BCL-2 and CCND1 3′UTRs containing binding sites for members of the miR-15 family and the consensus mimics were cloned from total RNA isolated from MSTO cells. The Promega MMLV RT kit was used to reverse transcribe 500 ng RNA, and 40 ng of the resultant cDNA was amplified using AmpliTaq Gold 360 (Promega) with specific forward and reverse primers. PCR amplicons were cloned into the TOPO TA vector (Thermo Scientific), the sequence confirmed by Sanger sequencing carried out at the Ramaciotti Centre (UNSW, Sydney), and subcloned into the pSiCheck2 plasmid (Promega). The resulting reporter constructs (1 µg), together with microRNA mimics or controls, were used to transfect 500,000 cells in 6-well plates. A dual luciferase assay (Promega) was carried out as per the manufacturer’s protocol 48 h after transfection.

### Tumour xenograft model

The effect of the consensus mimics on tumour growth in vivo was investigated in subcutaneous human xenograft models of PM and NSCLC in nude mice, as described previously [11]. Briefly, 1.5 × 10^6^ MSTO-211H or A549 cells in 50 µl serum-free medium mixed with 50 µl growth factor-reduced matrigel (BD Biosciences) were implanted in athymic (*nu*/*nu*) mice (4–6 weeks old; purchased from the Animal Resources Centre, Perth, Western Australia) via subcutaneous injection in the left flank. Tumour size was calculated by measuring length (*l*) and width (*w*) and calculating volume (*V* = *lw*^2^/2), with measurements obtained by an investigator blinded to the treatment groups. After the average tumour volume reached 100 mm^3^, mice were randomised into the indicated treatment groups. Mice were systemically administered with EGFR-targeted EDV^TM^ nanocells (EDVs) [32] loaded with con15/107.2 or control mimic via tail-vein injection 4 times per week. Tumour volume was measured at the indicated time points. All experiments were approved by the EnGeneIC Animal Ethics Committee and were performed in accordance with the relevant guidelines and regulations.

### In silico analysis of microRNA mimic binding to targets

To analyse binding the predicted binding of endogenous miR-16 and consensus mimics to targets in the 3′UTR of CCND1, the STarMir algorithm was used [33]. Consensus mimic sequences were manually entered, and those and the sequence of endogenous miR-16 were compared with the CCND1 RefSeq entry.

### Statistical analyses

Data are presented as mean ± SEM unless otherwise stated. Statistical analysis was carried out with Prism7.0 (GraphPad, La Jolla, CA, USA). A two-tailed unpaired *t*-test was used to test for significant differences for normally distributed data, and a two-tailed Mann–Whitney test was conducted on data that was not normally distributed. A one-way ANOVA test of variance was used to analyse proliferation assay data.

## Results

### Design of miR-15/107 consensus mimics

The miR-15/16 family consists of 8 members, all of which share an identical seed sequence (AGCAGC; nt 2–7) and homology over the remaining sequence ranging from 75 to 90%. Two additional microRNAs—miR-103 and miR-107—have the same AGCAGC sequence offset by 1 nucleotide (nt 1–6) and have been proposed as members of a larger miR-15/107 group [19]. Sequence alignment of the 7 microRNAs of this extended family previously found to be downregulated in PM cell lines and tumours [11] is shown in Fig. 1. As seen from this alignment there is considerable homology among all family members outside the seed region, with the exception of positions 11, 12, and 14–16.

As the predicted gene targets of the miR-15/107 group overlap but are not identical, we developed a series of non-natural microRNA mimics based on the consensus sequence. The consensus base for each position was called if it was identical in greater than 50% of the individual microRNAs. A single base was possible to call at all positions except 11 and 12, at which two bases had equal prominence. Thus, four related microRNA mimics were generated: 107.1, 107.2, 107.3 and 107.4 (Fig. 1C), each with varying homology to the miR-15/107 group.

### Consensus mimics have increased growth inhibitory activity in PM cells when compared with native miR-16

**Fig. 2:**
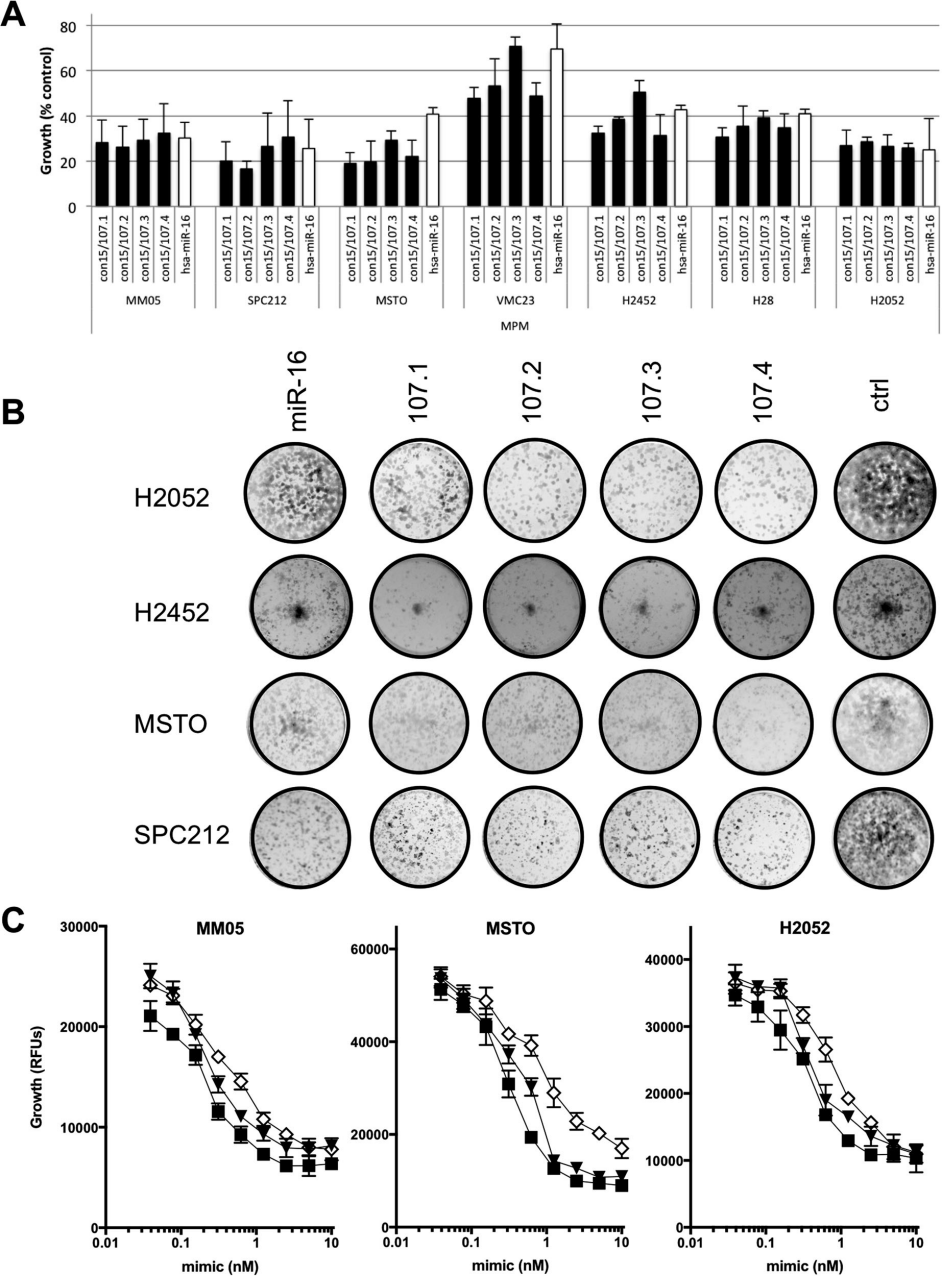
Consensus mimics inhibit growth in PM cell lines. **A** A panel of PM cell lines was transfected with microRNA mimics at a final concentration of 5 nM and proliferation was measured after 72 h. Data are mean ± SD (*n* = 3). **B** Cells transfected with the indicated microRNA mimics (5 nM) were transferred to 24-well plates and colony forming ability was measured after 7–10 days. A representative of three independent experiments is shown. **C** To determine the relative potency of the growth inhibitory activity of the mimics, cells were transfected with a microRNA mimics at a final concentration of 0.039 to 10 nM and proliferation was measured 96 h after transfection. Data are mean ± SD of triplicate measurements and are representative of three independent experiments.

Previously, we showed that restoring miR-15a, 15b and 16 levels in PM cell lines using mimics was able to inhibit cell growth [11]. To test whether the consensus mimics were able to inhibit growth in tumour cells, a panel of PM cell lines was transfected with these mimics as well as a mimic with sequence identical to native miR-16. Similar to the results obtained in our previous report, the growth of PM cell lines was inhibited by the native sequence (Fig. 2). When transfected at equimolar concentrations, the consensus mimics had equal or greater activity than the native sequence in both standard proliferation and colony formation assays in all cell lines tested (Fig. 2A, B). Expanding the range of mimic concentrations tested revealed that the consensus mimics were at least 2-fold more active than the native miR-16 sequence (Fig. 2C). Simultaneously restoring levels of miR-15a and miR-16 did not increase growth inhibition (data not shown).

### Consensus mimics are active in cell lines from other cancers types

**Fig. 3:**
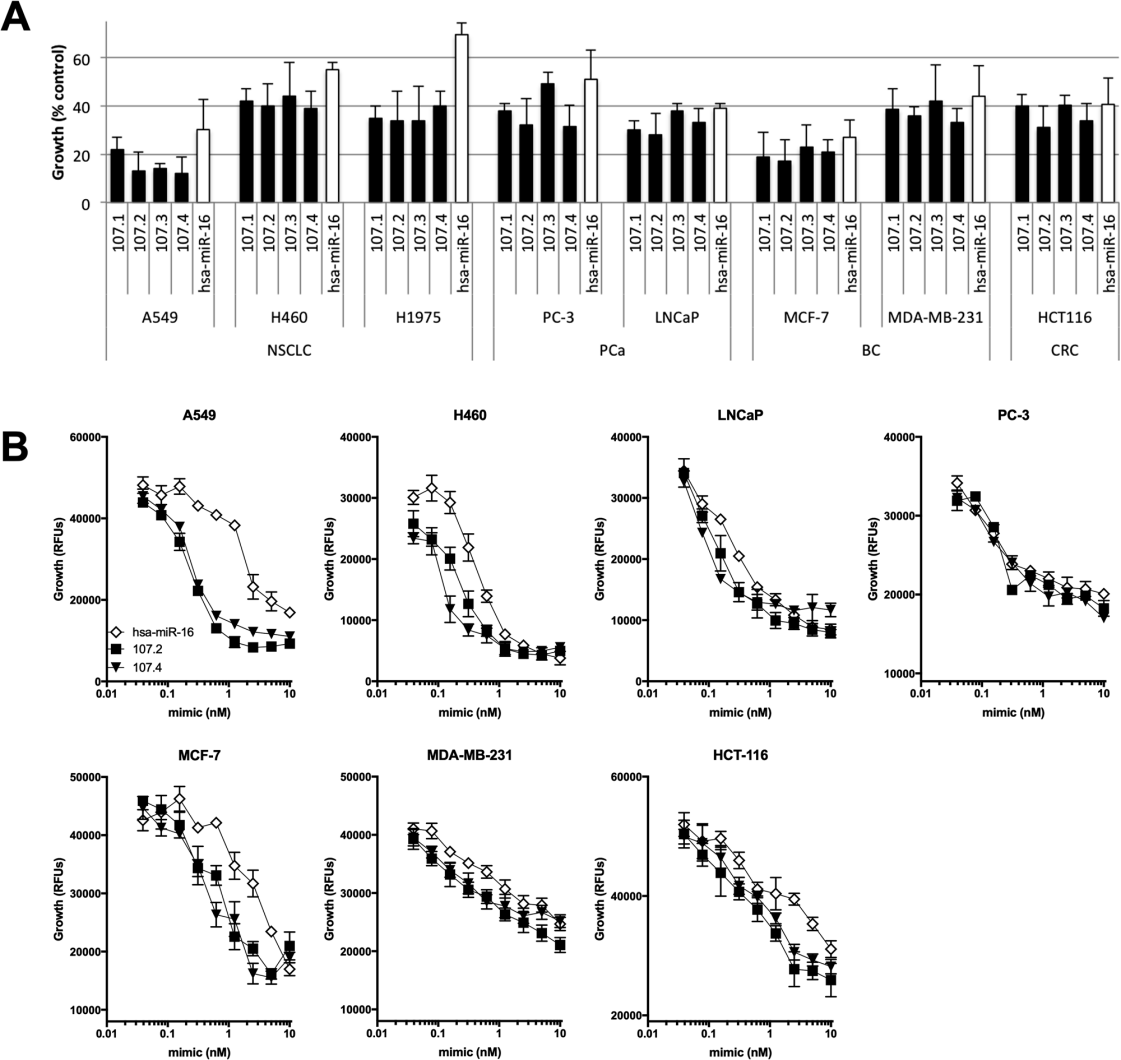
Activity of consensus mimics in other cancer cell types. **A** C lines derived from a range of tumour types were transfected with microRNA mimics at a final concentration of 5 nM and proliferation was measured after 72 h. Data are mean ± SD (*n* = 3). **B** The relative potency of the growth inhibitory activity of the mimics was determined as in Fig. 2. Data are mean ± SD of triplicate measurements and are representative of three independent experiments.

In addition to PM, NSCLC and prostate cancer are also frequently characterised by reduced expression of miR-15/16 [9–11], suggesting that the growth of cells lines derived from these tumours might also be susceptible to inhibition by the consensus mimics. We tested this by transfecting the NSCLC lines A549, H460 and H1975 and the prostate cancer lines PC-3 & LNCaP with the consensus mimics. Similar to the effects in PM cells, the consensus mimics were able to inhibit growth of these lines, and at concentrations at least 2-fold lower than those required for the native miR-16 sequence (Fig. 3). We next tested whether other cancers previously shown to respond to mimics corresponding to one or more miR-15 family members also responded to the consensus mimics. To test this, we transfected cell lines derived from breast (MCF-7 and MDA-MB-231) and colorectal cancer (HCT-116) with miR-16 and consensus mimics. Both breast cancer and colorectal cancer cell lines were strongly inhibited by transfection with miR-16 or the consensus mimics (Fig. 3A, B). In the case of the breast cancer cell lines, the growth inhibitory activity of the consensus mimics was again around two-fold more than that of the native sequence.

### Consensus mimics regulate similar targets to miR-16

**Fig. 4:**
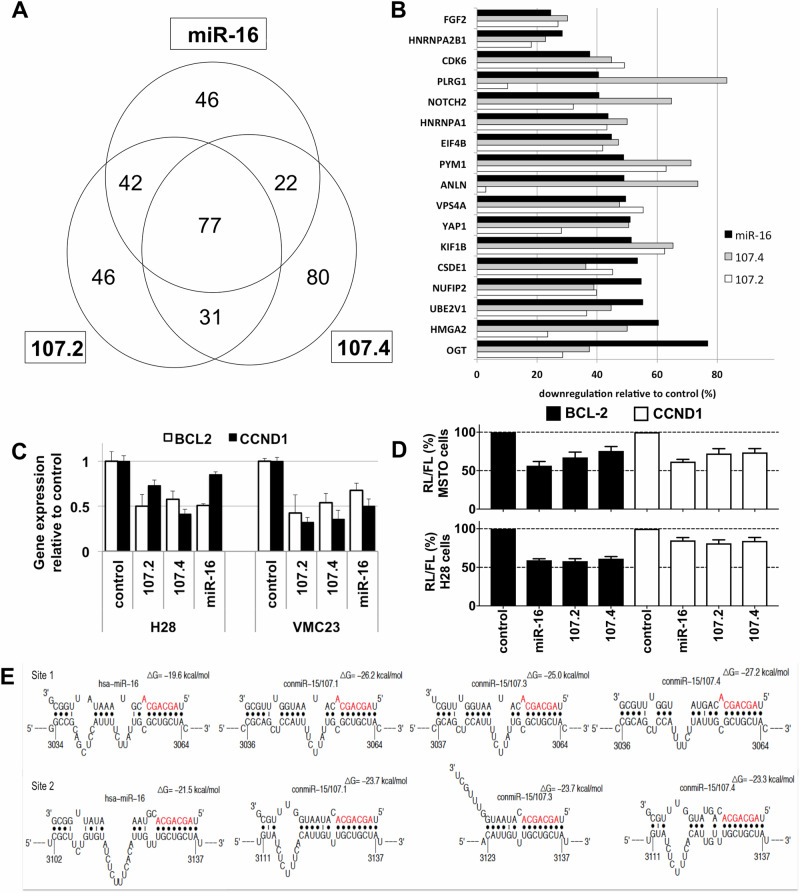
Effects of consensus mimics on gene expression. **A** Protein expression was measured in MSTO cells 72 h after transfection with miR-16 mimic, the consensus mimics 107.2 or 107.4 or a control mimic. The number of downregulated proteins following each treatment is shown. **B** SWATH-MS-based proteomic analysis of predicted miR-16 target genes that were downregulated in mimic transfected cells compared to controls. **C** The effect on target gene mRNA expression was measured 72 h after transfection of H28 or VMC23 cells with miR-16, the consensus mimics 107.2 or 107.4, or control mimic (5 nM). Data are mean ± SD (*n* = 3). **D** The binding of miR-16 and two consensus mimics to sites in target gene 3′UTRs was measured using luciferase reporter genes in MSTO and H28 cells. Data are mean ± SD of triplicate measurements and representative of three independent experiments. **E** Predicted binding sites and free energy calculations for miR-16 and the consensus mimics for 2 sites in the CCND1 3′UTR.

The consensus mimics 107.1, 107.2 and 107.4 had similar activity and were, in general, more active than 107.3, so we focused our additional experiments on 107.2 and 107.4. As the consensus mimics contain the same seed as the native miR-15 family members, they are predicted to have overlapping mRNA targets. Nevertheless, sequence features outside the seed can contribute to microRNA-mediated gene regulation [34, 35]. In order to determine whether differences in target genes were responsible for the increased activity of the consensus mimics, we carried out proteomic analysis using SWATH. We analysed protein expression in PM cell lines following transfection with a mimic corresponding to endogenous miR‐16 or consensus mimics 107.2 and 107.4. Similar numbers of targets were upregulated and downregulated in cells transfected with each of the three mimics compared with control mimic-transfected cells (Fig. 4A). Of the predicted targets of the miR-15 family downregulated in the SWATH analysis, the extent of downregulation was similar in all three treatments (Fig. 4B).

As the range of target genes of the consensus mimics appeared to be similar, we wondered whether the novel sequences were responsible for the increased activity of the consensus mimics. To determine the effect of mimics on target gene expression, we transfected PM cells with miR-16 and consensus mimics and carried out RT-qPCR on BCL2 and CCND1. Compared with miR-16, consensus mimics used at the same concentration reduced BCL2 mRNA levels to a similar extent but led to increased suppression of CCND1 mRNA expression (Fig. 4C). Interaction between the consensus mimics and the 3′UTRs of target genes was confirmed using luciferase assays in PM cell lines (Fig. 4D). In order to better understand the basis for this increased repression of CCND1 mRNA, we assessed the predicted binding of the various mimics to two target sites in the CCND1 3′UTR. This revealed a lower free energy associated with the binding of the consensus mimics to the target sites (Fig. 4E), indicative of enhanced binding.

### Consensus mimics sensitise tumour cells to gemcitabine

**Fig. 5:**
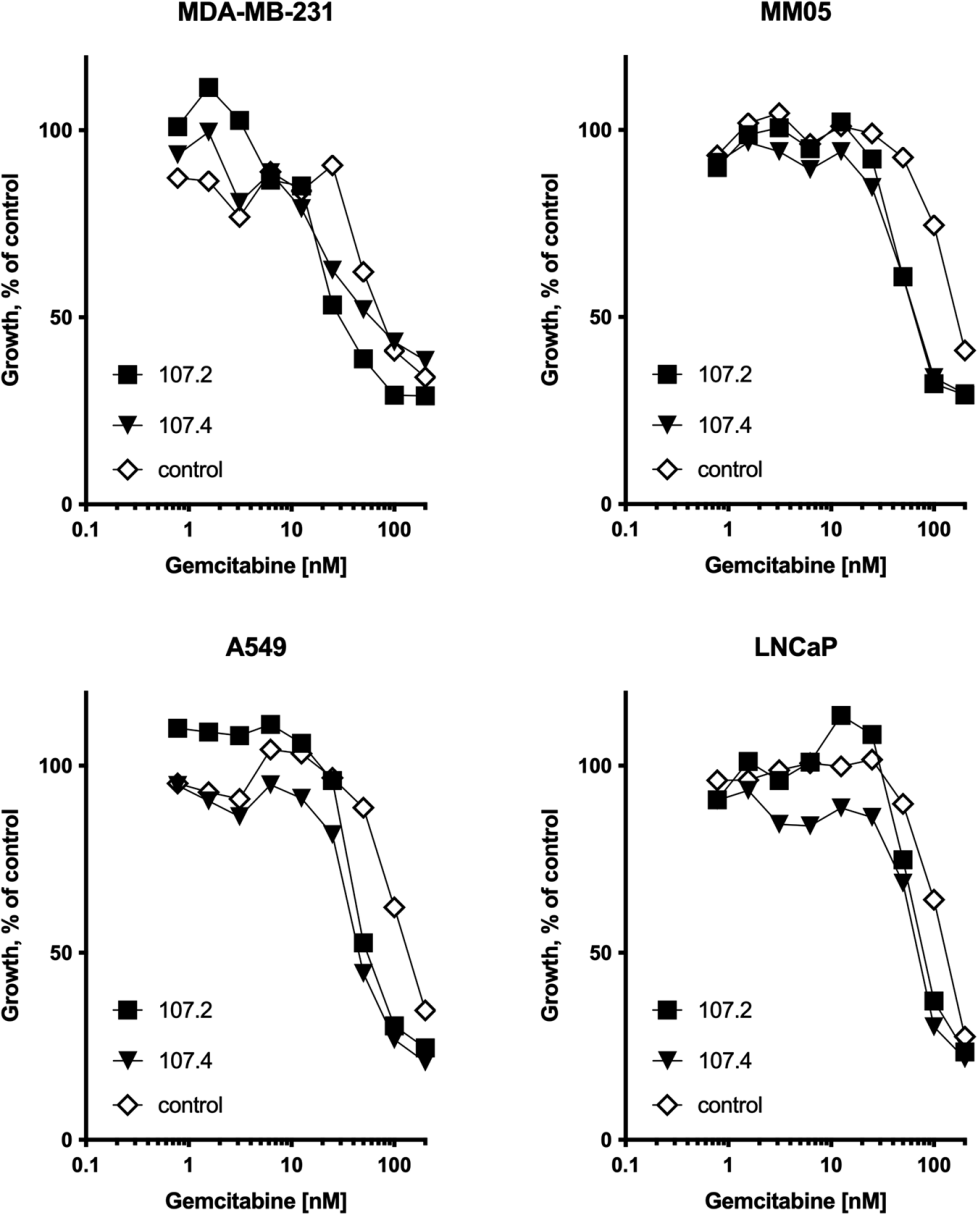
Effects of consensus mimics on sensitivity to gemcitabine. Cell lines were transfected with microRNA mimics at a final concentration of 5 nM, and gemcitabine at the indicated concentrations was added one day post-transfection. Proliferation was measured after 72 h. Data are mean ± SD of technical replicates (*n* = 3) and representative of three independent experiments producing similar results.

Previously, we demonstrated the ability of miR-16 to sensitise PM cell lines to gemcitabine and pemetrexed [11]. As gemcitabine is a component of the chemotherapy regimens used to treat a range of solid tumours, we investigated the effect of the consensus mimics on gemcitabine toxicity in cell lines of diverse origin. Compared with a control mimic, both 107.2 and 107.4 were able to increase the gemcitabine sensitivity of cell lines derived from PM, lung, breast and prostate cancer (Fig. 5).

### Consensus mimics are active in vivo

**Fig. 6:**
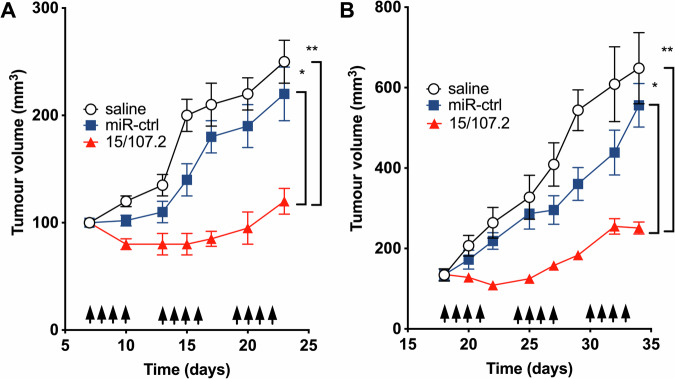
Consensus mimics control tumour growth in vivo. Xenograft tumours were formed by implantation of 1 × 10^6^ PM (**A**) or A549 (**B**) cells in nude mice. Once tumours reached a size of 100 mm^3^, treatment began. Mice were treated via tail-vein injection 4× per week (arrows) with 1 × 10^9^ EDVs containing the consensus mimic 107.2, control mimic, or saline. Tumour volume was measured on the indicated days. *P < 0.05, **P < 0.01. **C** Delivery of the consensus mimics to the xenografts was confirmed by the detection of the novel mimic sequences in RNA-seq analysis.

We next tested the activity of a consensus mimic conmiR-15/107.2 in vivo using xenograft models. To deliver the mimics systemically to tumour-bearing mice, we made use of the same EGFR antibody-targeted nanocell-based approach used in our previous studies [11, 36, 37]. Treating mice with conmiR-15/107.2-loaded minicells injected 4 times per week led to significant tumour growth inhibition in both xenograft models of mesothelioma (Fig. 6A) and non-small cell lung cancer (Fig. 6B) when compared with saline or control mimic-loaded minicells. These results are consistent with those obtained following the delivery of endogenous miR-16 in our previous study [11].

## Discussion

Since the discovery of their involvement in many aspects of cancer biology, much research has focused on developing microRNA-based therapeutics in cancer [38]. With global downregulation of microRNAs a typical feature of cancer [1], the replacement of lost tumour suppressor microRNAs has great potential to control dysregulated cancer cell growth and this is being investigated in phase I clinical trials [39]. The first microRNA mimic to enter the clinic was the MRX34, a liposome-formulated miR-34a mimic to treat liver cancer [40]. Following our pre-clinical studies of miR-16 restoration in PM [11], a phase I trial of a miR-15/16-based mimic for PM and NSCLC patients was completed. This trial included the first objective response in a patient treated with a microRNA-based drug [41], and the treatment was well tolerated [42]. The microRNA component of the treatment, dubbed TargomiRs, consisted of a mimic based on the consensus sequence derived from the miR-15/107 group of microRNAs. Here, we report on the design and pre-clinical development of these consensus mimics.

The consensus mimics were derived from alignment of the miR-15 family, along with the related miR-103 and miR-107. From this sequence, 4 mimics were designed to test the activity of the consensus sequence on cancer cell growth. While we are not aware of a similar approach from the literature, many mimics used previously in cancer research can be considered artificial to some extent, as they frequently consist of an active mature microRNA strand with its exact complement, rather than the natural sense strand, as this has been found to increase activity [43]. In addition, because the short seed sequence drives the majority of microRNA:mRNA interactions, this has been used to generate artificial microRNAs (a-miRs) targeting preselected genes of interest [44, 45]. This strategy has been used successfully to downregulate non-essential control genes [44] as well as genes upregulated in cancer [45].

The consensus mimics we generated frequently exhibited increased growth inhibitory activity compared with native microRNAs. This is somewhat surprising as both endogenous microRNAs and the synthetic sequences contain an identical seed sequence and would, therefore, be expected to share target mRNAs. While a comprehensive analysis of the differences in the targets of the consensus and endogenous microRNAs are beyond the scope of this study, previous studies suggest that alternative targeting mechanisms may lead to distinct repertoires of targets for the consensus mimics. Although most microRNA:mRNA interactions are governed by the binding of the seed sequence to its target [3], there are exceptions to this rule. Seedless sites have been shown to be involved in microRNA-mediated regulation of cell cycle genes [5] and G-bulge sites are common in mouse brains [4]. In addition, sites based on centred pairing have been demonstrated to repress a significant number of mRNA targets, particularly those without conventional seed sites [6].

In the mechanistic study of a-miRs designed to target preselected genes, features outside of the seed sequence were shown to be important factors in determining a-miR activity [44]. Analysis of the structure-activity relationships of over 200 engineered a-miRs with an identical seed sequence specific for target sites in the 3′UTR of the non-essential metabolic genes pyruvate carboxylase (PC) and glutaminase (GLS) revealed varying degrees of repression of reporter gene activity. Interestingly, the majority of a-miRs did not repress their targets, even when an exact seed match was present. In addition, binding of the more active a-miRs to their targets had significantly lower predicted hybridisation energy (indicating stronger binding) as well as Watson-Crick binding at the 3′-end [44]. These observations are consistent with the predicted binding of the consensus mimics used in our study with two important targets, BCL2 and CCND1, each of which had lower free energy when compared with the binding of native miR-16.

Proteomics analysis of a PM cell line transfected with consensus mimics and miR-16 revealed a number of targets—such as YAP1, OGT, Anilin, HMGA2 and CSDE1—which were previously shown (or are predicted) to be targeted by miR-15 or miR-16 in other cancer types [46–49]. It is possible that, in addition to those with well-known involvement in cancer biology such as BCL2 and CCND1, regulation of these genes is involved in the effect of the mimics on PM biology. Interestingly, cells treated with consensus mimics exhibited significant changes in the expression of multiple proteins in pathways associated with cell death. The majority of the genes were not predicted targets of the consensus mimics, at least when seed-based algorithms were used. These changes could, however, be related to alternative mechanisms of action such as centred pairing [6], which could create unique targets for the consensus mimics, as these vary most through the central portion and 3′-end. Whether the increased activity of the consensus mimics is due to alternative targets, enhanced hybridisation, or a combination of both, is the subject of continuing studies in our lab.

The consensus mimics and miR-16 were active in cell lines from a range of different tumour types. Those derived from PM, NSCLC and prostate cancer are from cancers well known (at the time of mimic design) to have reduced expression of miR-15 family members, and previous studies have demonstrated the tumour suppressor activity of the microRNAs in these cell lines [9–11]. In contrast, while there was less evidence at the time that they were consistently downregulated in breast and colorectal cancer, subsequent studies confirmed this [12, 14, 15], in line with the growth inhibitory activity we observed with our consensus mimics. In this latter case, increasing levels of miR-15 family microRNAs—and by extension use of consensus mimics sharing the same seed—in cells with normal expression of these microRNAs appears to have growth inhibitory effects. This is in line with previous studies using a miR-34a mimic, which was also shown to reduce growth independently of the expression level of this microRNA in the cells tested [50].

In addition to the growth inhibitory effects of the consensus mimics, they also sensitised cell lines to gemcitabine. These results are in line with our earlier report showing restoring miR-16 levels with a mimic sensitised PM cells to gemcitabine and pemetrexed [11]. As the consensus mimics in this study share the same seed and, therefore, predominantly target the same genes, this is likely related to the inhibition of BCL-2. A previous report showed that BCL-2 inhibition via antisense oligonucleotides (AS-ODN) led to increased gemcitabine sensitivity in PM [51] and transitional cell bladder carcinoma [52], as did the reduction in BCL-2 expression following treatment of PM cells with eIF4E-specific AS-ODNs [53]. Similarly, reduced BCL-2 expression following treatment with short interfering RNAs (siRNAs) restored gemcitabine sensitivity in pancreatic cancer cells in vitro [54] and in vivo [55]. Interestingly, the relationship between miR-16, BCL-2 and gemcitabine activity is supported by studies with curcumin, the active component of the spice turmeric. Curcumin increased gemcitabine activity in pancreatic cancer models in part by inhibiting the expression of BCL-2 and other anti-apoptotic proteins [56] and was shown to increase levels of miR-15a and miR-16 in breast cancer cells [57].

In the last 10 years, numerous studies investigating systemic delivery of microRNA mimics have been carried out in animal models. Our current study used EDV nanocells [32] to deliver microRNA mimics to cancer cells via systemic administration. Similar to the results of our previous studies focusing on miR-16 [11] and miR193a-3p [37] in PM, and miR-7 in adrenocortical cancer [36], mice injected with EGFR-targeted minicells loaded with consensus mimic inhibited tumour growth significantly more than minicells carrying control mimic. All of these studies were carried out with 5 × 10^9^ EDVs, containing ~1.5 µg microRNA mimic, which is considerably lower than concentrations used in comparable preclinical studies [40, 58–61]. In addition, preliminary results from the recently completed phase I trial in PM patients using the same dose suggest that this amount of microRNA is safe and may be sufficient to induce clinical effects in humans [42]. It is interesting to note that the trial of MRX34 [62]—in which patients received considerably greater doses of microRNA mimic—was terminated in September 2016 due to severe adverse events. Whether this difference is due to the higher dose of RNA remains to be determined, but it is well known that dsRNA induces inflammatory reactions via interaction with Toll-like receptors [63]. It is possible that new strategies to chemically modify microRNA mimics may ameliorate the inflammatory response to prevent adverse events [58].

In conclusion, our study provides evidence that a consensus mimic based on the miR-15/107 group exhibited enhanced growth activity in comparison with a mimic based on the endogenous miR-16 sequence. Our phase I clinical trial in thoracic cancer (NCT02369198) made use of a microRNA mimic with consensus sequence, and although a phase I trial, demonstrated clear signs of activity [41, 42, 64]. The data presented here suggest that a similar strategy would be potentially effective in other cancer types.

## Data Availability

Source data and reagents are available from the corresponding author upon reasonable request.
